# TRPM7 and TRPM8 Ion Channels in Pancreatic Adenocarcinoma: Potential Roles as Cancer Biomarkers and Targets

**DOI:** 10.6064/2012/415158

**Published:** 2012-07-19

**Authors:** Nelson S. Yee, Ada S. Chan, Julian D. Yee, Rosemary K. Yee

**Affiliations:** ^1^Division of Hematology-Oncology, Department of Medicine, Penn State College of Medicine, Penn State Hershey Cancer Institute, Penn State Milton S. Hershey Medical Center, Pennsylvania State University, Hershey, PA 17033-0850, USA; ^2^Penn State Harrisburg School of Humanities, Pennsylvania State University, Middletown, PA 17057, USA

## Abstract

Transient receptor potential (TRP) ion channels are essential for normal functions and health by acting as molecular sensors and transducing various stimuli into cellular and physiological responses. Growing evidence has revealed that TRP ion channels play important roles in a wide range of human diseases, including malignancies. In light of recent discoveries, it has been found that TRP melastatin-subfamily members, TRPM7 and TRPM8, are required for normal and cancerous development of exocrine pancreas. We are currently investigating the mechanisms which mediate the functional roles of TRPM7 and TRPM8 and attempting to develop these ion channels as clinical biomarkers and therapeutic targets for achieving the goal of personalized therapy in pancreatic cancer.

## 1. Introduction

The purpose of this paper is to examine the newfound roles of the transient receptor potential (TRP) ion channels, in particular the melastatin subfamily members TRPM7 and TRPM8, in pancreatic cancer and discuss their potential as clinical biomarkers and therapeutic targets. Pancreatic adenocarcinoma is a highly lethal disease; its incidence has been rising and the prognosis remains dismal [1]. In order to develop effective interventions for prevention, early detection, and treatment of this malignant disease, it is critical to identify the etiologic risk factors and understand the mechanism underlying formation and progression of pancreatic neoplasia [2]. Steady progress has been made in advancing knowledge in molecular biology and genetics of pancreatic development under normal and malignant conditions [2, 3]. Accumulating evidence has implicated that ion channels are important modulators of the pathogenic mechanisms underlying many human diseases such as cancer [4]. There is a paucity of knowledge about the role of ion channels in normal pancreatic development and during malignant transformation. By using the zebrafish model, we have discovered the developmental role of Trpm7 in exocrine pancreas [5]. Moreover, we have demonstrated the novel roles of the human orthologue TRPM7 and its sub-family member TRPM8 in pancreatic adenocarcinoma [5–8]. 

From embryogenesis to adult life, ion channels have been shown to play crucial roles in regulating the diverse cellular processes and physiological functions. Aberrant expression and/or activities of ion channels have been implicated in various disease states, including cancer. Ion channels, particularly the TRP family members, detect a wide range of stimuli in the cellular microenvironment [9]. These include light, changes in temperature and osmolarity, pain, mechanical stress, touch, acidic and alkaline pH, and taste. Functional TRP channels are assembled as tetramers, which consist of four identical TRP subunits (homotetramers) or similar TRP subunits (heterotetramers). Each TRP subunit contains six transmembrane segments S1 to S6, with the amino- and carboxyl-terminals facing the intracellular side of the membrane. Voltage sensing has been shown to occur in the region between segments S1 and S4, and the hydrophobic central pore through which ions flow is located between the 5th and 6th segments [10]. The TRP channels exhibit relative ionic selectivity, and they exert regulatory effects on cellular proliferation, survival, differentiation, and migration by maintaining cationic homeostasis and modulating signaling pathways [11]. There is a growing body of evidence indicating that aberrant expression of some of the TRP channels contributes to the uncontrolled proliferation and growth of malignant tumors [4].

In the TRP family of ion channels, there are seven TRP subfamilies including TRPC (classical or canonical), TRPM (melastatin), TRPA (ankyrin), TRPV (vanilloid), TRPP (polycystin), TRPN (NOMP-C homologues), and TRPML (mucolipin). These TRP subfamilies of channels share common gross architecture, and each subfamily is characterized by unique structural features [10]. TRPM represents one of the largest and most diverse TRP subfamily, and there are eight members in the TRPM subfamily. The TRPM channels possess the TRP domain (also conserved in the TRPC and TRPN subfamilies), which is a conserved 25-amino acid sequence distal to the S6 region and proximal to the carboxyl-terminus. It has been proposed that the TRP domain contributes to tetramerization of the channel subunits and also gating of the channel. The TRPM channels are nonselectively permeable for cations, and they play diverse physiological roles by sensing physical and chemical stimuli in the microenvironment and responding through modulation of the intracellular ionic levels and signaling pathways. Accumulating evidence from morphological, biochemical, and genetic studies has begun to elucidate the functional roles of the TRPM channels in various aspects of human malignancies. Until recently, it was unknown if the TRPM channels had any influential role in pancreatic cancer. Using a combination by positional cloning and candidate testing, we discovered that the zebrafish *sweetbread (swd)* mutations affect *Trpm7*, and we identified a novel role of Trpm7 in controlling the size of exocrine pancreas through regulation of cell cycle progression and growth in pancreatic epithelia [5]. In attempt to test the hypothesis that deregulated expression and/or activities of developmental regulators contributes to carcinogenesis, we have discovered that TRPM7 and TRPM8 are aberrantly overexpressed in pancreatic adenocarcinoma and required for proliferation of the cancer cells [5–8]. These studies provide a novel link between cellular sensation and signal modulation in pancreatic cancer. With the goal of identifying and developing clinical biomarkers and therapeutic targets in pancreatic cancer, we are actively investigating the mechanistic roles of TRPM7 and TRPM8 ion channels in the initiation, growth, and invasion of pancreatic neoplasia.

## 2. TRPM7 Channel Kinase: Physiological Functions and Roles in Development and Cancer 

TRPM7 is a divalent cation-permeable channel with an intrinsic kinase, and it carries out its physiological functions by acting as a cellular sensor and signal transducer through its ion channel and kinase activity [12–15]. Among the TRP family of ion channels, only the TRPM7 channel and its closest homologue TRPM6 possess such protein kinase domain. The function of the TRPM7 channel requires the activity of its endogenous kinase, which is capable of phosphorylating the serine and threonine residues of protein [13, 14]. While interaction of the TRP domain of TRPM7 with phosphatidylinositol-4,5-bisphosphate (PIP_2_) leads to activation of the TRPM7 channel, stimulation of phospholipase C (PLC) results in hydrolysis of PIP_2_ and thus inactivation of the TRPM7 channel [15]. Moreover, interaction between the kinase domain and PLC regulates the channel activity of TRPM7 in a manner that is dependent on cAMP and protein kinase A [16]. TRPM7 kinase also has autophosphorylating ability, which is regulated by intracellular level of Mg^2+^-ATP [16–19]. TRPM7 is ubiquitously expressed, and it controls cellular homeostasis of ions, particularly Mg^2+^ and Ca^2+^, and modulates the signaling pathways involved in cell cycle progression, cytodifferentiation, survival, and migration. 

TRPM7 plays a regulatory role in a variety of cellular effects, which are dependent on the cell types. These include cell survival in lymphocytes, neurons, and mast cells [12, 20, 21], proliferation of osteoblasts and pancreatic epithelia and cancer cells [5–8, 22, 23]; cytodifferentiation in T-lymphocytes [24]. Moreover, TRPM7 has been shown to regulate cell adhesion in neuroblastoma [25], cell volume in epithelia of kidney and cervical uterus [26], and cell migration in osteoblasts and fibroblasts [23, 27]. Electrophysiological data indicate that cellular influx of Mg^2+^ through the TRPM7 channel results in changes of intracellular concentrations of Mg^2+^ or Ca^2+^. Hypothetically, the resulting perturbed ionic homeostasis leads to modulation of the signaling complex and produces various cellular responses of TRPM7 [14, 15, 20, 27].

In vertebrates, the functional roles of TRPM7 are organ-specific, as revealed by genetic studies in model organisms. This is illustrated by the requirement of Trpm7 in skeletal formation and skin pigmentation in zebrafish. Moreover, it has been shown that Trpm7 is required for proliferation and survival of melanoblasts and osteoblasts [5, 28–30]. In mice, genetic deletion of *Trpm7* produces lethality in the embryos, and disruption of *Trpm7* in lymphoid tissues impairs lymphocytic development [24]. Recently, we have reported that the zebrafish* sweetbread *(*swd*) mutations affect *Trpm7*, the zebrafish orthologue of mammalian *TRPM7* [5]. From a genome-wide ethylnitrosourea-induced mutagenesis screen for mutations affecting the exocrine pancreas, we previously identified and recovered the zebrafish *swd* alleles, *swd* ^*p*75*fm*^, and *swd* ^*p*82*mf*^ [2, 30]. Besides, genetic deletions in *Trpm7* were identified in the zebrafish *touchtone* (*tct*) mutants, which have reduced skin pigmentation and abnormally developed skeletons [28]. Examination of the *trpm*7^*swd*^ and *trpm*7^*tct*^ mutant larvae revealed that their exocrine pancreata are relatively small [2, 5, 30]. Consistent with this finding, the pancreatic acini and ducts in the mutants are hypomorphic; however, specification of cell fate and cytodifferentiation in exocrine pancreatic epithelia appear normal [2, 5, 30].

By impairing cell cycle progression and cell growth, the *trpm*7^*swd*^ and *trpm*7^*tct*^ mutations cause diminished proliferation of exocrine pancreatic epithelia [5]. Consistent with the role of TRPM7 as a transporter of Mg^2+^, it was demonstrated that the impaired growth of exocrine pancreas in the *trpm*7^*swd*^ and *trpm*7^*tct*^ mutants can be partially improved by adding extra Mg^2+^ to the embryo medium. The supplementary Mg^2+^-induced partial rescue of the exocrine pancreatic phenotype in the *trpm7* mutants is associated with repression of the mRNA levels of *p*21^*cdkn*1*a*^ and *cyclin G1* [5]. Furthermore, the *trpm*7^*swd*^ and *trpm*7^*tct*^ mutant larvae express elevated levels of *suppressor of cytokine signaling 3a* (*socs3a*), a negative regulator of epidermal growth factor (EGF) receptor-mediated signaling [5]. Exocrine pancreatic growth in the *trpm7* mutants can be rescued by repression of *socs3a* by either supplementary Mg^2+^ or antisense oligos directed against *socs3a* mRNA. These effects are associated with improved cell cycle progression and cell growth in the exocrine pancreatic epithelia [5]. These studies support a novel role of Trpm7 in exocrine pancreas during early development, and the proliferative role of Trpm7 in exocrine pancreatic epithelia is mediated by Mg^2+^-sensitive mechanisms that involve Socs3a.

**Figure 1:**
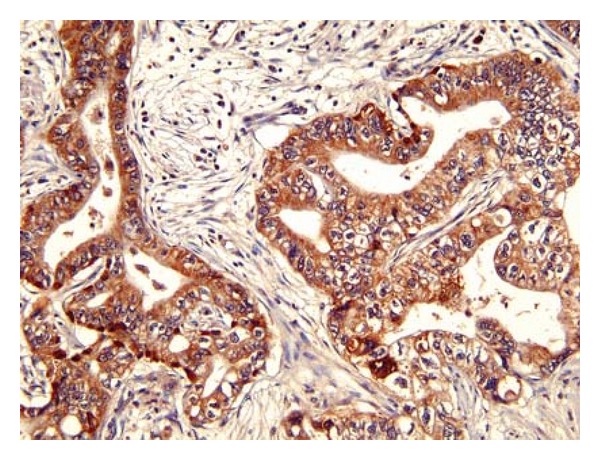
Over-expression of TRPM7 channel in human pancreatic adenocarcinoma. Anti-TRPM7 immunoreactivity in pancreatic tissues was analyzed using TRPM7-specific antibodies, and the reaction was detected by using 3,3′-diaminobenzidine followed by counterstaining with hematoxylin. The brown color indicates expression of TRPM7 protein in pancreatic adenocarcinoma. Control for immunohistochemistry includes consecutive histological sections from the same paraffinized block being processed in parallel without anti-TRPM7 antibodies. Relative low level of anti-TRPM7 immunoreactivity was detected in the controls (data not shown).

By translating the developmental studies of Trpm7 in zebrafish to humans, we have found the previously unidentified role of TRPM7 in pancreatic adenocarcinoma. In human pancreatic adenocarcinoma tissues, TRPM7 protein is aberrantly overexpressed [5] (Figure 1). Similarly, the mRNA levels of *TRPM7* are elevated in the majority of human pancreatic adenocarcinoma cell lines being examined [5]. By using small interfering RNA (siRNA) to inhibit translation of *TRPM7* mRNA in the pancreatic cancer cells, we provide evidence that TRPM7 is required for cellular proliferation by preventing cell cycle arrest in the G_0_/G_1_ phases [5]. In the TRPM7-deficient cells, the reduced proportions of cells in the S and G_2_/M phases correlate with upregulated expression of the cyclin-dependent kinase inhibitor *p*21^*CDKN*1*A*^ and repression of *cyclins G1* and* B1 *[5]. Consistent with the findings in zebrafish *TRPM7* mutants and in agreement with the role of TRPM7 as a transporter or divalent cations, addition of extra Mg^2+^ in the culture medium rescues the proliferative defect of TRPM7-deficient pancreatic cancer cells [5].

In a recent report, we have demonstrated a novel role of TRPM7 in preventing replicative senescence. RNA interference-mediated silencing of TRPM7 did not cause an increase in apoptotic cell death, as analyzed by flow cytometry [7]. The pancreatic cancer cells with silenced expression of *TRPM7* exhibit morphological features including enlarged cell size and multiple nuclei, suggestive of replicative senescence. This was confirmed by assaying for senescence associated *β*-galactosidase (SA *β*-gal) activity in the TRPM7-deficient cells, which were shown to have undergone accelerated cellular senescence [7]. Besides, our current studies using human pancreatic adenocarcinoma cells provide novel evidence that silenced expression of *TRPM7* impedes cell migration (Yee NS, unpublished data), suggesting TRPM7 plays an important role in invasion and metastasis of pancreatic cancer.

**Figure 2:**
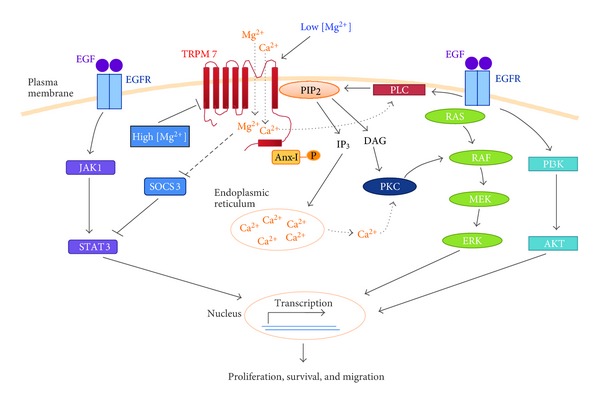
A working model for the signaling mechanisms that mediate the cellular functions of TRPM7 in pancreatic adenocarcinoma. Low intracellular level of Mg^2+^ or phosphatidylinositol-4,5-bis-phosphate (PIP_2_) activates the ion channel of TRPM7. Once activated, TRPM7 allows inflow of Mg^2+^ and/or Ca^2+^from the extracellular medium into the cytosol. The rise of intracellular Mg^2+^ leads to activation of intracellular reactions that result in cellular proliferation, survival, and migration. On the other hand, the Ca^2+^ influx leads to activation of Ca^2+^-sensitive phospholipase C (PLC) and hydrolysis of PIP_2_, thus providing negative feedback inhibition of TRPM7 activity. Hydrolysis of PIP_2_ produces inositol-1,4,5-triphosphate (IP_3_), which triggers intracellular Ca^2+^ release and produces diacylglycerol (DAG). The increased Ca^2+^ or DAG activates protein kinase C (PKC). PKC in turn activates RAF in the RAS/ERK pathway, leading to transcription of a variety of genes and resulting in cellular proliferation, survival, and migration.

Taken together, TRPM7 is required for Mg^2+^-dependent cellular proliferation in the developing exocrine pancreas and in pancreatic adenocarcinoma through modulation of the cell cycle regulators. Our current research focuses on the signaling mechanisms including the Socs3a pathway that mediate the functional roles of TRPM7 in exocrine pancreatic organogenesis as well as in the growth and invasion of pancreatic neoplasia. We hypothesize that aberrantly up-regulated expression of TRPM7 in pancreatic adenocarcinoma contributes to the uncontrolled proliferation and invasion of the cancer cells by facilitating cell cycle progression and cell migration and by preventing replicative senescence. In our working model, activation of TRPM7 by various stimuli modulates intracellular Mg^2+^ and/or Ca^2+^ levels and interact with the epidermal growth factors (EGF-) induced signaling pathways, resulting in cell cycle progression, cell survival, and cell migration in pancreatic adenocarcinoma (Figure 2). Besides pancreatic adenocarcinoma, the role of TRPM7 has also been implicated in cancers that arise in other organs, such as retinoblastoma [31], head and neck carcinoma [32], gastric carcinoma [33], breast carcinoma [34], and nasopharyngeal carcinoma [35]. While the mechanism underlying the aberrant overexpression of TRPM7 in malignant neoplasia has yet to be determined, the existing data support the exploration of its clinical significance in pancreatic adenocarcinoma and the other malignancies.

## 3. TRPM8 Ion Channel: Physiological Functions and Roles in Cancer

With identification of the role of TRPM7 in pancreatic development and cancer, we examined the expression of the other TRPM subfamily of ion channels in a panel of human pancreatic adenocarcinoma cell lines. We discovered that TRPM8, a nonselective, voltage-gated, and Ca^2+^ permeable ion channel is aberrantly expressed with a functional role in pancreatic cancer [6, 8]. For the TRPM8 channel to be functional, the coiled coil domain and a region between the amino acids 40 and 86 of the amino-terminus are essential [36]. Both the amino- and carboxyl-terminal regions are dispensable for assembly of the channel tetramer and its trafficking to the membrane [36]. Cold temperature (15°C to 25°C) or application of cooling compounds (such as menthol, eucalyptol, and icilin) has been shown to activate the TRPM8 channel, leading to an increase in intracellular concentration of Ca^2+^ [37, 38]. Indeed, the TRPM8 channel is permeable to both monovalent ions (e.g., Na^+^, K^+^) and divalent ions (e.g., Ca^2+^, Ba^2+^). Opening of the TRPM8 channel is dependent on voltage, and the probability of channel opening is enhanced with depolarization of the membrane.

**Figure 3:**
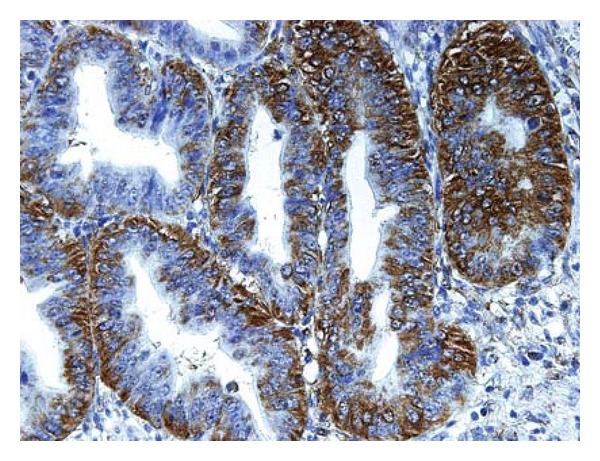
Aberrant expression of TRPM8 channels in human pancreatic adenocarcinoma. Anti-TRPM8 antibodies were employed for immunohistochemical analysis of pancreatic tissues, followed by color reactions using 3,3′-diaminobenzidine and counterstaining with hematoxylin. Expression of TRPM8 protein in pancreatic adenocarcinoma is indicated by the brown color. Consecutive histological sections from the same paraffinized block were processed in parallel in the absence of anti-TRPM8 antibodies as control, and there is no specific anti-TRPM8 immunoreactivity being detected (data not shown).

TRPM8 is selectively expressed in human adult tissues. A relatively high level of *TRPM8* mRNA is present in the prostate gland [39] and a discernible level in the liver, dorsal root ganglion, and trigeminal ganglion neurons [40]. Aberrant expression of TRPM8 has been reported in neoplastic tissues, some of which include prostate carcinoma, breast adenocarcinoma, lung cancer, colorectal cancer, melanoma, urinary bladder cancer, neuroblastoma, neuroendocrine tumor [41–47], and in the most recent finding pancreatic adenocarcinoma [6, 8]. In a panel of seven human pancreatic adenocarcinoma cell lines, expression of TRPM8 is consistently up-regulated, as determined by real-time polymerase chain reaction and compared to the immortalized pancreatic ductal epithelia [6]. Analysis of human pancreatic adenocarcinoma tissues by immunohistochemistry using specific anti-TRPM8 antibodies shows that TRPM8 is aberrantly expressed [6, 8] (Figure 3). For comparison, there is no detectable immunoreactivity against TRPM8 in normal or nonmalignant pancreatic ducts [6].

The aberrant expression of TRPM8 in pancreatic adenocarcinoma is associated with a function role. SiRNA-mediated silencing of *TRPM8* in pancreatic cancer cells reduced their ability to proliferate and progress through the cell cycle [6]. Such proliferative defect is associated with elevated mRNA levels of the cyclin-dependent kinase inhibitors *p*21^*CDKN*1*A*^ and *p*27^*CDKN*1*B*^ [6]. Moreover, phase contrast microscopy and DAPI staining revealed that TRPM8-deficient cells are enlarged and flat, containing multiple nuclei and cytoplasmic vacuoles [6]. These morphological features suggest that the TRPM8-deficient cells underwent nonapoptotic cell death via replicative senescence. This hypothesis was tested by repressing *TRPM8* with siRNA and then assaying for activity of SA *β*-gal as a marker of cellular senescence. Result of this study indicates that SA *β*-gal is activated in TRPM8-deficient cells but not in the cells treated with nontargeting control siRNA [8]. 

**Figure 4:**
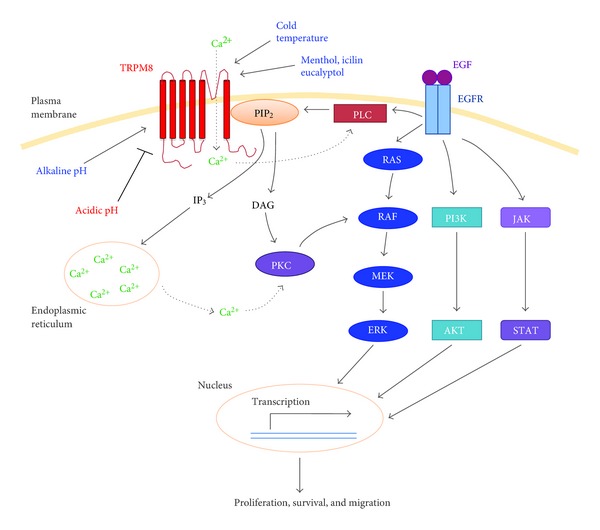
A working model for the proliferative, prosurvival, and migratory roles of TRPM8 in pancreatic adenocarcinoma cells. The ion channel activity of TRPM8 can be stimulated by cold temperature, menthol, alkaline pH, or phosphatidylinositol-4,5-bis-phosphate (PIP_2_), but inhibited by acidic pH. Once stimulated, the TRPM8 channel allows cellular influx of Ca^2+^, which in turn leads to activation of Ca^2+^-sensitive phospholipase C (PLC) and hydrolysis of PIP_2_. These events provide negative feedback inhibition of the TRPM8 channel activity and also produce inositol-1,4,5-triphosphate (IP_3_). As a consequence, Ca^2+^ is released from the intracellular stores and diacylglycerol (DAG) generated. The elevated Ca^2+^ or DAG activates protein kinase C (PKC) and thus RAF in the RAS/ERK pathway. These signaling events culminate in transcription of proliferative, prosurvival, and promigratory genes.

Taken together, the TRPM8 channel is required for cellular proliferation in pancreatic adenocarcinoma by preventing cell cycle arrest and replicative senescence. Although it is unclear how TRPM8 becomes aberrantly expressed in pancreatic adenocarcinoma, current data suggest that TRPM8 plays a functional role in pancreatic tumor by contributing to its uncontrolled growth and progression. We propose a working model that will form the basis for testing our hypotheses regarding the signaling mechanisms that mediate the proliferative and prosurvival effects of TRPM8 in pancreatic adenocarcinoma by using cultured cells and animal models (Figure 4).

## 4. Potential Roles of TRPM7 and TRPM8 as Biomarkersand Targets for Personalized Therapies in Pancreatic Cancer

**Figure 5:**
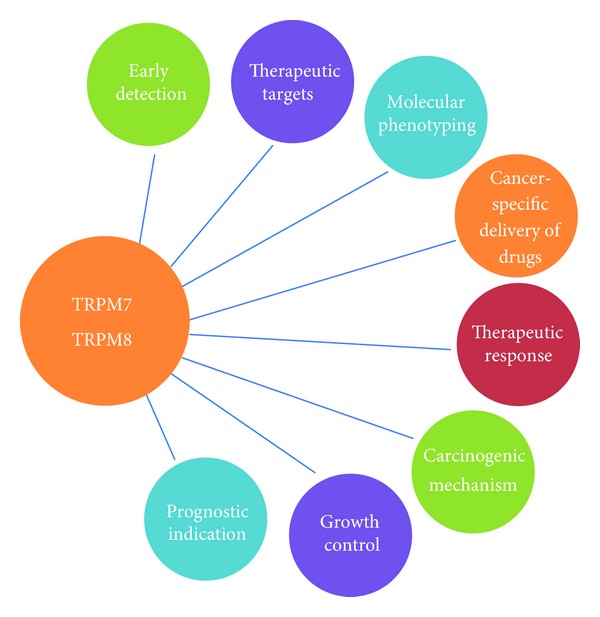
A schematic diagram to illustrate the diverse roles of TRPM7 and TRPM8 channels as potential clinical biomarkers and therapeutic targets in pancreatic adenocarcinoma, and also as growth and migratory regulators in normal exocrine pancreas and in malignant neoplasia of pancreas. The various roles of TRPM7 and TRPM8 are likely applicable to other malignant tumors that exhibit aberrant expression and/or activities of TRPM7 and TRPM8.

The aberrant over-expression and proliferative roles of the TRPM7 and TRPM8 ion channels in pancreatic adenocarcinoma suggest a unique opportunity of their development as clinical biomarkers and therapeutic targets (Figure 5). As potential biomarkers of pancreatic cancer, TRPM7 and TRPM8 channels can be exploited to characterize the molecular phenotype of tumors, to facilitate early detection of primary and metastatic tumors, to monitor and predict treatment responses, to determine patient prognosis, and to aid targeted delivery of cancer-specific therapeutics. As a potential therapeutic approach in pancreatic adenocarcinoma, we can target the TRPM7 and TRPM8 ion channels via chemical and/or genetic modulation of their channel activities and their associated signaling pathways. Such approach can also be pursued in combination with cytotoxic drugs [7] or other molecularly targeted agents. Therefore, immense potential exists for TRPM7 and TRPM8 channels to be developed as clinically useful biomarkers and valid therapeutic targets with the hope of accomplishing personalized therapy for patients with pancreatic adenocarcinoma and other malignant diseases [48].

## 5. Summary and Prospective

The novel findings of Trpm7 ion channel in the growth control of exocrine pancreas in zebrafish prompted discovery of the aberrant expression as well as the proliferative and migratory roles of TRPM7 and TRPM8 channels in human pancreatic adenocarcinoma. Such experimental evidence supports the idea that developmental regulators play important roles in oncogenesis [2, 30, 49–54] and provide a novel link of cellular sensors to pancreatic cancer. The focus of our basic research studies is to determine the signaling mechanisms that mediate the proliferative and migratory roles of TRPM7 and TRPM8 in pancreatic epithelia and cancer cells. These results are expected to provide mechanistic insights into pancreatic carcinogenesis and generate new hypotheses regarding the impact of physical and chemical stimuli on the formation and progression of pancreatic neoplasia.

The findings of TRPM ion channels in pancreatic development and adenocarcinoma also provide support for translation of developmental modulators of exocrine pancreas into tumor biomarkers and molecular targets for therapy [2, 51, 55, 56]. The focuses of our translational research studies are (i) to develop TRPM7 and TRPM8 channels as clinical biomarkers, with the goals of improving molecular phenotype of pancreatic tumor and predicting their response to treatment and (ii) to exploit TRPM7 and TRPM8 channels as therapeutic targets and also as ligands for directing tumor-specific delivery of anticancer therapeutics. We hope that the complimentary approaches used in our studies will ultimately lead to developing novel and effective personalized strategies for improving treatment of patients with pancreatic cancer and other malignant tumors.

## References

[1] Siegel R, Naishadham D, Jemal A (2012). Cancer statistics, 2012. *Cancer Journal for Clinicians*.

[2] Yee NS, Han H, Grippo PJ (2010). Zebrafish as a biological system for identifying and evaluating therapeutic targets and compounds. *Drug Discovery in Pancreatic Cancer: Models and Techniques*.

[3] Hidalgo M (2010). Pancreatic cancer. *The New England Journal of Medicine*.

[4] Lehen’kyi V, Prevarskaya N (2011). Oncogenic TRP channels. *Advances in Experimental Medicine and Biology*.

[5] Yee NS, Zhou W, Liang IC (2011). Transient receptor potential ion channel Trpm7 regulates exocrine pancreatic epithelial proliferation by Mg^2+^-sensitive Socs3a signaling in development and cancer. *Disease Models and Mechanisms*.

[6] Yee NS, Zhou W, Lee M (2010). Transient receptor potential channel TRPM8 is over-expressed and required for cellular proliferation in pancreatic adenocarcinoma. *Cancer Letters*.

[7] Yee NS, Zhou W, Lee M, Yee RK (2012). Targeted silencing of TRPM7 ion channel induces replicative senescence and produces enhanced cytotoxicity with gemcitabine in pancreatic adenocarcinoma. *Cancer Letters*.

[8] Yee NS, Brown RD, Lee MS (2012). TRPM8 ion channel is aberrantly expressed and required for preventing replicative senescence in pancreatic adenocarcinoma: potential role of TRPM8 as a biomarker and target. *Cancer Biology and Therapy*.

[9] Nilius B, Owsianik G (2011). The transient receptor potential family of ion channels. *Genome Biology*.

[10] Latorre R, Zaelzer C, Brauchi S (2009). Structure-functional intimacies of transient receptor potential channels. *Quarterly Reviews of Biophysics*.

[11] Dadon D, Minke B (2010). Cellular functions of transient receptor potential channels. *International Journal of Biochemistry and Cell Biology*.

[12] Nadler MJS, Hermosura MC, Inabe K (2001). LTRPC7 is a Mg*·*ATP-regulated divalent cation channel required for cell viability. *Nature*.

[13] Runnels LW, Yue L, Clapham DE (2001). TRP-PLIK, a bifunctional protein with kinase and ion channel activities. *Science*.

[14] Schmitz C, Perraud AL, Johnson CO (2003). Regulation of vertebrate cellular Mg^2+^ homeostasis by TRPM7. *Cell*.

[15] Runnels LW, Yue L, Clapham DE (2002). The TRPM7 channel is inactivated by PIP2 hydrolysis. *Nature Cell Biology*.

[16] Takezawa R, Schmitz C, Demeuse P, Scharenberg AM, Penner R, Fleig A (2004). Receptor-mediated regulation of the TRPM7 channel through its endogenous protein kinase domain. *Proceedings of the National Academy of Sciences of the United States of America*.

[17] Ryazanova LV, Dorovkov MV, Ansari A, Ryazanov AG (2004). Characterization of the protein kinase activity of trpm7/chak1, a protein kinase fused to the transient receptor potential ion channel. *Journal of Biological Chemistry*.

[18] Matsushita M, Kozak JA, Shimizu Y (2005). Channel function is dissociated from the intrinsic kinase activity and autophosphorylation of TRPMT/ChaK1. *Journal of Biological Chemistry*.

[19] Demeuse P, Penner R, Fleig A (2006). TRPM7 channel is regulated by magnesium nucleotides via its kinase domain. *Journal of General Physiology*.

[20] Aarts M, Iihara K, Wei WL (2003). A key role for TRPM7 channels in anoxic neuronal death. *Cell*.

[21] Wykes RCE, Lee M, Duffy SM, Yang W, Seward EP, Bradding P (2007). Functional transient receptor potential melastatin 7 channels are critical for human mast cell survival. *Journal of Immunology*.

[22] Abed E, Moreau R (2007). Importance of melastatin-like transient receptor potential 7 and cations (magnesium, calcium) in human osteoblast-like cell proliferation. *Cell Proliferation*.

[23] Abed E, Moreau R (2009). Importance of melastatin-like transient receptor potential 7 and magnesium in the stimulation of osteoblast proliferation and migration by platelet-derived growth factor. *American Journal of Physiology*.

[24] Jin J, Desai BN, Navarro B, Donovan A, Andrews NC, Clapham DE (2008). Deletion of Trpm7 disrupts embryonic development and thymopoiesis without altering Mg^2+^ homeostasis. *Science*.

[25] Clark K, Langeslag M, van Leeuwen B (2006). TRPM7, a novel regulator of actomyosin contractility and cell adhesion. *The EMBO Journal*.

[26] Numata T, Shimizu T, Okada Y (2007). TRPM7 is a stretch- and swelling-activated cation channel involved in volume regulation in human epithelial cells. *American Journal of Physiology*.

[27] Wei C, Wang X, Chen M, Ouyang K, Song LS, Cheng H (2009). Calcium flickers steer cell migration. *Nature*.

[28] Elizondo MR, Arduini BL, Paulsen J (2005). Defective skeletogenesis with kidney stone formation in dwarf zebrafish mutant for trpm7. *Current Biology*.

[29] McNeill MS, Paulsen J, Bonde G, Burnight E, Hsu MY, Cornell RA (2007). Cell death of melanophores in zebrafish trpm7 mutant embryos depends on melanin synthesis. *Journal of Investigative Dermatology*.

[30] Yee NS, Lorent K, Pack M (2005). Exocrine pancreas development in zebrafish. *Developmental Biology*.

[31] Hanano T, Hara Y, Shi J (2004). Involvement of TRPM7 in cell growth as a spontaneously activated Ca^2+^ entry pathway in human retinoblastoma cells. *Journal of Pharmacological Sciences*.

[32] Jiang J, Li MH, Inoue K, Chu XP, Seeds J, Xiong ZG (2007). Transient receptor potential melastatin 7-like current in human head and neck carcinoma cells: role in cell proliferation. *Cancer Research*.

[33] Kim BJ, Park EJ, Lee JH, Jeon JH, Kim SJ, So I (2008). Suppression of transient receptor potential melastatin 7 channel induces cell death in gastric cancer. *Cancer Science*.

[34] Guilbert A, Gautier M, Dhennin-Duthille I, Haren N, Sevestre H, Ouadid-Ahidouch H (2009). Evidence that TRPM7 is required for breast cancer cell proliferation. *American Journal of Physiology*.

[35] Chen JP, Luan Y, You CX, Chen XH, Luo RC, Li R (2010). TRPM7 regulates the migration of human nasopharyngeal carcinoma cell by mediating Ca^2+^ influx. *Cell Calcium*.

[36] Phelps CB, Gaudet R (2007). The role of the N terminus and transmembrane domain of TRPM8 in channel localization and tetramerization. *Journal of Biological Chemistry*.

[37] McKemy DD, Neuhausser WM, Julius D (2002). Identification of a cold receptor reveals a general role for TRP channels in thermosensation. *Nature*.

[38] Peier AM, Moqrich A, Hergarden AC (2002). A TRP channel that senses cold stimuli and menthol. *Cell*.

[39] Fonfria E, Murdock PR, Cusdin FS, Benham CD, Kelsell RE, McNulty S (2006). Tissue distribution profiles of the human TRPM cation channel family. *Journal of Receptors and Signal Transduction*.

[40] Takashima Y, Daniels RL, Knowlton W, Teng J, Liman ER, McKemy DD (2007). Diversity in the neural circuitry of cold sensing revealed by genetic axonal labeling of transient receptor potential melastatin 8 neurons. *Journal of Neuroscience*.

[41] Tsavaler L, Shapero MH, Morkowski S, Laus R (2001). Trp-p8, a novel prostate-specific gene, is up-regulated in prostate cancer and other malignancies and shares high homology with transient receptor potential calcium channel proteins. *Cancer Research*.

[42] Mergler S, Strowski MZ, Kaiser S (2007). Transient receptor potential channel TRPM8 agonists stimulate calcium influx and neurotensin secretion in neuroendocrine tumor cells. *Neuroendocrinology*.

[43] Yamamura H, Ugawa S, Ueda T, Morita A, Shimada S (2008). TRPM8 activation suppresses cellular viability in human melanoma. *American Journal of Physiology*.

[44] Li Q, Wang X, Yang Z, Wang B, Li S (2009). Menthol induces cell death via the TRPM8 channel in the human bladder cancer cell line T24. *Oncology*.

[45] Louhivuori LM, Bart G, Larsson KP (2009). Differentiation dependent expression of TRPA1 and TRPM8 channels in IMR-32 human neuroblastoma cells. *Journal of Cellular Physiology*.

[46] Wondergem R, Bartley JW (2009). Menthol increases human glioblastoma intracellular Ca^2+^, BK channel activity and cell migration. *Journal of Biomedical Science*.

[47] Chodon D, Guilbert A, Dhennin-Duthille I (2010). Estrogen regulation of TRPM8 expression in breast cancer cells. *BMC Cancer*.

[48] Yee NS Targeting the molecular phenotype of pancreatic cancer toward the goal of patient-tailored therapy.

[49] Yee NS, Yusuff S, Pack M (2001). Zebrafish pdx1 morphant displays defects in pancreas development and digestive organ chirality, and potentially identifies a multipotent pancreas progenitor cell. *Genesis*.

[50] Yee NS, Furth EE, Pack M (2003). Clinicopathologic and molecular features of pancreatic adenocarcinoma associated with Peutz-Jeghers syndrome. *Cancer Biology and Therapy*.

[51] Yee NS, Pack M (2005). Zebrafish as a model for pancreatic cancer research. *Methods in Molecular Medicine*.

[52] Yee NS, Gong W, Huang Y (2007). Mutation of RNA Pol III subunit rpc2/polr3b leads to deficiency of subunit Rpc11 and disrupts zebrafish digestive development. *PLoS Biology*.

[53] Chun SG, Yee NS (2010). Werner’s syndrome as a hereditary risk factor for exocrine pancreatic cancer: potential role of WRN in pancreatic tumorigenesis and patient-tailored therapy. *Cancer Biology and Therapy*.

[54] Zhou W, Liang IC, Yee NS (2011). Histone deacetylase 1 is required for exocrine pancreatic epithelial proliferation in development and cancer. *Cancer Biology and Therapy*.

[55] Chun SG, Zhou W, Yee NS (2009). Combined targeting of histone deacetylases and hedgehog signaling enhances cytoxicity in pancreatic cancer. *Cancer Biology & Therapy*.

[56] Yee NS, Zhou W, Chun SG, Liang -C I, Yee RK (2012). Targeting developmental regulators of zebrafish exocrine pancreas as a therapeutic approach in human pancreatic cancer. *Biology Open*.

